# Different Statistical Approaches to Characterization of Adipocyte Size in Offspring of Obese Rats: Effects of Maternal or Offspring Exercise Intervention

**DOI:** 10.3389/fphys.2018.01571

**Published:** 2018-11-15

**Authors:** Carlos A. Ibáñez, Magaly Vázquez-Martínez, J. Carlos León-Contreras, Luis A. Reyes-Castro, Guadalupe L. Rodríguez-González, Claudia J. Bautista, Peter W. Nathanielsz, Elena Zambrano

**Affiliations:** ^1^Reproductive Biology Department, Instituto Nacional de Ciencias Médicas y Nutrición Salvador Zubirán, Mexico City, Mexico; ^2^Experimental Patology Section, Instituto Nacional de Ciencias Médicas y Nutrición Salvador Zubirán, Mexico City, Mexico; ^3^Department of Animal Science, University of Wyoming, Laramie WY, United States

**Keywords:** adipocyte, adipocyte size distribution, adipose tissue, maternal obesity, exercise intervention

## Abstract

Adipocyte size (AS) shows asymmetric distribution related to current metabolic state, e.g., adipogenesis or lipolysis. We profiled AS distribution using different statistical approaches in offspring (F1) of control (C) and obese (MO) mothers (F0) with and without F0 or F1 exercise. Offspring from F0 exercise were designated CF0ex and MOF0ex. Exercised F1 of sedentary mothers were designated CF1ex and MOF1ex. F1 retroperitoneal fat cross-sectional AS was measured by median, cumulative distributions, data dispersion and extreme values based on gamma distribution modeling. F1 metabolic parameters: body weight, retroperitoneal fat, adiposity index (AI), serum leptin, triglycerides (TG) and insulin resistance index (IRI) were measured. Male and female F1 AS showed different cumulative distribution between C and MO (*p* < 0.0001) therefore comparisons were performed among C, CF0ex and CF1ex groups and MO, MOF0ex and MOF1ex groups. MO AI was higher than C (*p* < 0.05) and male MOF1ex AI lower than MO (*p* < 0.05). Median AS was higher in male and female MO vs. C (*p* < 0.05). Male and female MOF0ex and MOF1ex reduced median AS (*p* < 0.05). Lower AS dispersion was observed in male CF1ex and MOF1ex vs. CF0ex and MOF0ex, respectively. MO reduced small and increased large adipocyte proportions vs. C (*p* < 0.05); MOF0ex increased small and MOF1ex the proportion of large adipocytes vs. MO (*p* < 0.05). MOF0ex reduced male IRI and female TG vs. MO (*p* < 0.05). MOF1ex reduced male and female leptin (*p* < 0.05); CF1ex reduced male leptin (*p* < 0.05). Conclusions: several factors, diet, physical activity and gender modify AS distribution. Conventional AS distribution methods normally do not include analyzes of extreme, large and small adipocytes, which characterize different phenotypes. Maternal high fat diet affects F1 AS distribution, which was programmed during development. F0ex and F1ex have gender specific F1 beneficial effects. AS distribution characterization helps explain adipose tissue metabolic changes in different physiological conditions and will aid design of efficacious interventions to prevent and/or recuperate adverse developmental programming outcomes. Finally, precise identification of effects of specific interventions as exercise of F0 and/or F1 are needed to improve outcomes in obese women and their obesity prone offspring.

## Introduction

We (Zambrano et al., 2010, 2016; Rodriguez et al., 2012; Santos et al., 2015; Vega et al., 2015; Bautista et al., 2017) and others (Sen and Simmons, 2010; Elshenawy and Simmons, 2016; Lecoutre et al., 2016; Wankhade et al., 2016) have studied the rat as an experimental animal model of the metabolic consequences of maternal (F0) obesity (MO) in offspring (F1). In addition, several studies have shown differential responses in male and female F1 from obese mothers (Vega et al., 2015; Almeida et al., 2017; Carrillo et al., 2017). Variable physiological conditions can profoundly either reduce or increase adipose tissue lipid storage capacity by up to 15-fold (Berry et al., 2013). Adipose tissue volume expansion depends on two mechanisms: hypertrophy [increased adipocyte size (AS)] and/or hyperplasia (increased adipocyte number) (Ali et al., 2013; Cohen and Spiegelman, 2016). Increased AS has been related to pathogenesis of insulin resistance, dyslipidemia and impaired adipokine secretion (Murphy et al., 2017) and represents an easily quantifiable marker of adipose tissue dysfunction that can be compared across pathologies (Jung and Choi, 2014).

Adipocytes isolated by collagenase digestion can be accurately analyzed by Coulter counting or flow cytometry (Hirsch and Gallian, 1968; McLaughlin et al., 2014; Fang et al., 2015). However, this approach has many disadvantages due to the potential cell damage by protease activity, incomplete cell separation and lack of specificity of automatic particle detection. In addition, AS obtained from isolated adipocytes is often reported as multimodal histograms (Bradshaw et al., 2003), whose statistical distribution is frequently empirically modeled by sophisticated differential equations (Jo et al., 2009, 2010, 2013), which potentially discourage their generalized use.

Histological determination of AS has many advantages over isolated cell methods in terms of specificity of adipocyte detection, since cell morphology and structure are better conserved by paraformaldehyde fixation and paraffin embedding (Parlee et al., 2014). Moreover, adipocyte boundaries, can be easily delimited in a digitalized microscope field and the area enclosed by each individual cell can be accurately quantified with common image analysis software (Berry et al., 2014).

Due to high AS heterogeneity, there is currently no single generally accepted method for size comparison and analysis. For example, we (Zambrano et al., 2010), and others (Chen and Farese, 2002; Bringhenti et al., 2011) have generally compared mean AS by the commonest parametric methods. Nevertheless, AS in several conditions cannot be analyzed under the assumption of a normal distribution. Consequently, AS median size is usually compared by non-parametric statistics test (Berends et al., 2013), and descriptive statistics are employed to show AS differences at extreme values. Many published papers only address changes in larger cells (Laye et al., 2009; Jo et al., 2010; Baik et al., 2014; Fukuda-Tsuru et al., 2014).

Since an increased proportion of large adipocytes is associated with increased differentiation rates (Camp et al., 2002; McLaughlin et al., 2007; Skurk et al., 2007); and increased proportion of small adipocytes is associated with augmented proliferative rate (Kajita et al., 2012), it has become of interest to determine AS distribution between large and small sized adipocytes under several physiologic conditions to elucidate the cellular mechanisms involved in expansion of specific fat depots. Thus, it is likely that different proportions of small, medium and large adipocytes will provide insight into different cellular mechanisms of adipose tissue expansion. It has been proposed that “*cross-sectional static cell-size distributions for a range of snapshots of animal development can be used to deduce the dynamics of adipose tissue growth, if we can appropriately analyze the snapshots with the help of mathematical modeling*” (Jo et al., 2009).

In this report, several approaches are proposed to characterize the AS distribution in terms of AS central tendency (represented either as mean or median), measures of data dispersion and extreme values of AS. Measures regarding data spread and data distribution shape were estimated under gamma distribution assumption. Adipocyte boundaries in a histological slide resemble a tiling composed of polygons with all the vertices pointing outward similar to a Poisson-Voronoi diagram, thereby allowing the use of gamma distribution as a statistical model (Tanemura, 2003).

We studied our well-established rat model of increased F1 adiposity programmed by F0 obesity to characterize the AS distribution by different analytical approaches. Since F0 (Vega et al., 2015) and F1 (Santos et al., 2015) exercise interventions (F0ex and F1ex) lead to different F1 metabolic benefits, we hypothesize that different AS distribution will be observed depending on the nature of the exercise intervention.

## Materials and Methods

### Ethical Approval

This study was carried out in accordance with the recommendations of Mexican law on animal protection (NOM-062-ZOO-1999). The protocol (BRE-112/CINVA 271) was approved by Animal Experimentation Ethics Committee of the Instituto Nacional de Ciencias Médicas y Nutrición Salvador Zubirán (INCMNSZ). Female albino Wistar rats were born and maintained in the animal facility of the INNCMSZ, accredited by the Association for Assessment and Accreditation of Laboratory Animal Care International (AAALAC).

### Experimental Design

Rats were maintained under controlled lighting (lights on from 07:00 to 19:00 h at 22–23°C) and fed normal laboratory chow (Zeigler Rodent RQ22-5, United States) containing 22.0% protein, 5.0% fat, 31.0% polysaccharide, 31.0% simple sugars, 4.0% fiber, 6.0% minerals and 1.0% vitamins (w/w), energy 4.0 kcal g^-1^. Between 16 and 17 weeks of age, when they weighed 200–240 g, females were bred to randomly assigned, non-litter mate, proven male breeders. At delivery, on day 0, litters that provided Founder Generation (F0) mothers were culled to 10 pups, each containing at least four females. At weaning (day 21) one female F0 pup from each litter was randomly assigned to either a control (C; *n* = 16) group fed laboratory chow or to a maternal obesity group (MO; *n* = 16) fed a high energy, obesogenic diet containing 23.5% protein, 20.0% animal lard, 5.0% fat, 20.2% polysaccharide, 20.2% simple sugars, 5.0% fiber, 5.0% mineral mix, 1.0% vitamin mix (w/w), energy 4.9 kcal g^-1^; diet contents, including vitamin and mineral mix were according to the recommendation for rodents of the American Institute of Nutrition/AIN-93G (Reeves et al., 1993). Thus, each F0 group contained only one female from any litter and F0 females in different groups, but not within groups, were sisters, providing homogeneity in F0 mothers’ own developmental programming and genetics.

**FIGURE 1:**
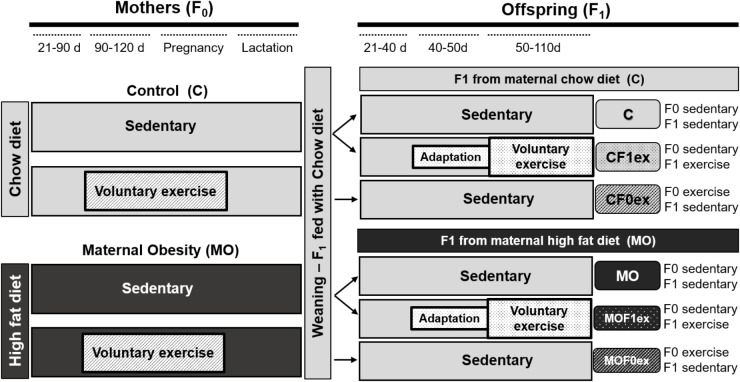
Experimental design. Timeline for study on maternal (F0) or offspring (F1) exercise intervention. Control (C); Maternal Obesity (MO); days (d); F1 from maternal exercise intervention were called CF0ex and MOF0ex, whilst F1 from offspring exercise intervention were denoted as CF1ex and MOF1ex.

At day 90, 1 month before breeding, one half of C and MO F0 were randomly selected to continue their diet and begin voluntary wheel-running exercise at (C exercised—CF0ex; MO exercised—MOF0ex). The opportunity to wheel run was available through pregnancy. Remaining females continued with their respective diets during pregnancy and lactation (Figure 1).

F0 female rats were placed with proven male breeders on day 120 and conceived during the next cycle. The day in which spermatozoa were detected in a vaginal smear was designed as day of conception—day 0. To minimize consumption of the high energy-obesogenic diet by the males during the mating period, males were placed with females at night and removed each morning. Male breeders did not perform any exercise. Lactating mothers were maintained on their pregnancy diet. Litter size and pup weight were recorded at birth. F1 anogenital distance was measured to identify males and females (Zambrano et al., 2006). To ensure F1 homogeneity, on postnatal day (PND) 2, all litters studied were adjusted to 10 pups with equal numbers of males and females as closely as possible. F1 were weaned at PND 21, housed five per cage and fed Chow diet throughout the study. At PND 50 eight males and eight females from C and MO sedentary mothers from different litters were randomly selected to begin voluntary wheel-running exercise (C exercised—CF1ex; MO exercised—MOF1ex). F1 from maternal exercise intervention were called CF0ex and MOF0ex, whilst F1 from offspring exercise intervention were denoted as CF1ex and MOF1ex (Figure 1).

### F0 Voluntary Exercise

CF0ex and MOF0ex rats were adapted to wheel run on 2 days on the week before day 90 allowing the rats to be in contact with the rodent wheel in 15-min sessions. From day 90 to breeding at 120 were trained five times a week following a schedule established in a previous study and consisted of a 15-min run followed by a 15-min rest and a second 15-min run between 9:00 and 12:00 h (Vega et al., 2016). F0 rats during pregnancy ran for only one 15-min session five times a week. Voluntary exercise varied in late gestation, some animals did not run the day before parturition while others completed the schedule until parturition. Mothers did not exercise while nursing.

### F1 Voluntary Exercise

CF1ex and MOF1ex rats were adapted in the same way, allowing them to be in contact with the rodent wheel 2 days in the week before PND 50. F1 rats ran 15 min which they always completed, followed by 15-min rest and a second 15-min run 5 days per week for 2 months (PND 50–PND 110). The last bout of exercise took place 24 h prior tissue collection.

### F1 Sampling

At PND 110, following a 6-h fast, F1 rats were euthanized under general anesthesia with isoflurane exposure and rapid decapitation performed by trained personnel, experienced in using a rodent guillotine (Thomas Scientific, United States) between 12:00 and 14:00 h. Trunk blood was collected, and serum separated and preserved at -70°C until biochemical and hormonal analysis. Visceral fat depots located inside the thorax (mediastinal) and abdomen (omental, perirenal, retroperitoneal, epididymal, periovarian, perivesical and parametrial) were excised and weighed. Adiposity index (AI) was calculated as 100 × total adipose tissue (g)/body weight (g). The retroperitoneal fat pad was fixed in 10% paraformaldehyde, dehydrated and paraffin-embedded (Vankelecom, 2009).

### F1 Cross-Sectional AS

A total of 5 μm thickness paraffin-embedded retroperitoneal fat sections were mounted on poly-L-lysine pre-coated slides. After deparaffinization and rehydration slides were stained with hematoxylin and eosin (Cardiff et al., 2014). We analyzed at least 25 different cells per animal, using an Olympus BX51 light microscope (Melville, NY, United States) at 20× magnification. AS was measured manually by delimiting the adipocyte cross-sectional area in digital images using AxioVisio LE software real 4.8 version (Zeiss^®^ copyright 2006–2010 Stuttgart-Germany) in at least 180 cells per group corresponding to an average of 25 cells per animal. AS was reported as the area obtained in μm^2^. All histological measurements were performed by an observer blinded to the nature of the tissue source.

### Characterization of Offspring AS Distribution by Different Statistical Approaches

#### AS Central Tendency and Data Distribution

Mean and median AS differences among groups were compared. In addition, AS data from all animals were visualized in grouped scatter plots and data dispersion analyzed by plotting the median vs. interdecile range.

#### AS Distribution Comparisons

Data distribution differences in AS were assessed by statistical comparison of their cumulative distributions.

#### AS Gamma Distribution Modeling

**FIGURE 2:**
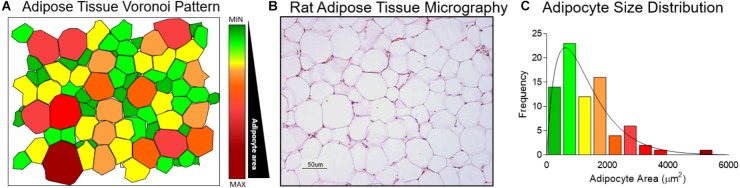
Adipocyte size gamma distribution modeling. **(A)** Cross sectional view of adipose tissue represented as a Voronoi Diagram. **(B)** Wistar rat retroperitoneal fat depot histologic slide stained with haematoxylin and eosin at 20× magnification. **(C)** Adipocyte size distribution determined in the Voronoi Diagram.

Normal distribution was evaluated using the AS sample mean and standard deviation. Since a cross-sectional view of adipose tissue emulates a Poisson-Voronoi diagram (Figure 2A) and the AS in a histological slide (Figure 2B) show an asymmetric distribution (Figure 2C).

Histograms of AS relative frequency with area intervals, approximated by the Sturges rule (Scott, 2009) of 500 μm^2^, (i.e., 0–500 μm^2^, 501–1,000 μm^2^, 1,001–1,500 μm^2^…until the maximal observed area interval), were overlaid with their corresponding gamma probability density function. The determination coefficient (R^2^) was calculated for theoretical normal and gamma quantiles by Q-Q plot analyses. Shape (mean/SD)^2^ and scale [(SD)^2^/mean] were estimated individually for each rat, to calculate the small and large AS proportions.

#### Estimation of Small and Large AS Proportions

Small and large adipocyte cut-off points were defined, respectively, by the 10th and 90th percentile of the gamma distribution of the C group and analyses by non-parametric comparisons.

### Blood Measurements

Glucose and triglycerides (TG) were analyzed enzymatically in a Synchron CX auto analyzer (Beckman Coulter, Co.); insulin and leptin were determined by radioimmunoassay. Insulin Resistence Index (IRI) was calculated from IRI = glucose (mmol/L) × insulin (μU/mL) × 22.5^-1^ (Vega et al., 2015).

### Statistical Analysis

One male and one female per litter were chosen randomly to provide *n* = 8 rats per sex per group. Shape, rate, and scale parameter are reported as median with interquartile range. Body weight, AI and blood measurements are reported as mean ± SEM.

Adipocyte size cumulative distributions by the two sample Kolmogorov–Smirnov test (Graph Pad Prism 6.0) and the observed *p*-value was adjusted with the Bonferroni correction to counteract three sample simultaneous comparisons.

To assess the effect of F0 diet on F1 phenotype, we compared F1 parameters of C and MO by Student’s *t*-test for normally distributed data (body weight, AI and blood measurements) or the Mann–Whitney *U* test for non-normally distributed data (AS distribution). To assess effects of F0ex and F1ex within C and MO, we performed one-way ANOVA for normally distributed data and Kruskal–Wallis test for non-normally distributed data. AS dispersion of extreme values were determined by gamma distribution probability comparison. *p* < 0.05 was considered statistically different.

## Results

### F1 Body Weight and AI at PND 110

**FIGURE 3:**
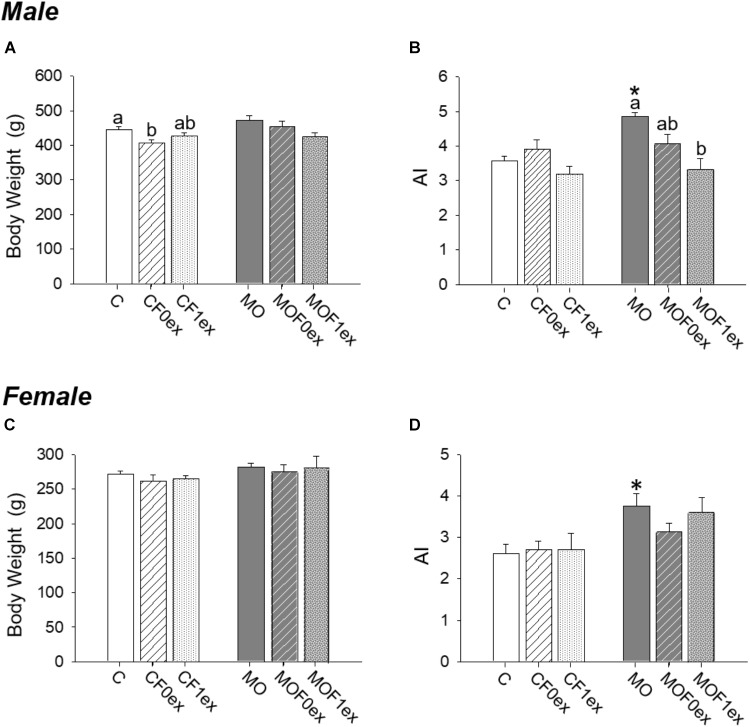
Offspring at 110 days. Offspring body weight **(A,C)** and adiposity index **(B,D)** for males and females, respectively. Data are mean ± SEM, *n* = 8 rats per group; ^∗^*p* < 0.05 vs. MO and for groups not sharing at least one letter (a, b) in the same maternal diet (C, CF0ex and CF1ex) or (MO, MOF0ex and MOF1ex).

F1 body weight was similar between C and MO within males and females (Figures 3A,C). Male body weight was lower in CF0ex than C but similar in CF1ex. In the female F1, F0ex or F1ex did not affect body weight (Figure 3C). AI was higher in MO when compared to C (*p* < 0.05) for both males and females (Figures 3B,D). AI was similar in all control groups in both males and females. MOF0ex AI was lower than MO (Figure 3B). No differences were observed in the female AI (Figure 3D).

### Characterization of Offspring AS Distribution

#### AS Central Tendency and Data Distribution

**FIGURE 4:**
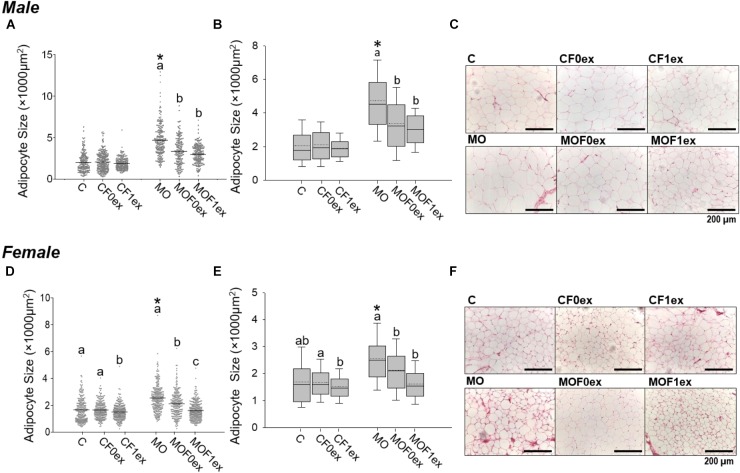
Adipocyte size central tendency in offspring at 110 days. Male and Female F1 mean adipocyte size and data dispersion **(A,D)**. Median (solid line) and mean (doted line) adipocyte size with interquartile range **(B,E)**. Representative photomicrograph of retroperitoneal adipose tissue H&E stained and observed at 20× magnification. **(C,F)** for male and female, respectively. *n* = 179–250 adipocytes from eight rats per group. ^∗^*p* < 0.05 vs. MO and for groups not sharing at least one letter (a, b) in the same maternal diet (C, CF0ex, and CF1ex) or (MO, MOF0ex, and MOF1ex).

Mean AS was higher in MO compared to C (*p* < 0.05) for both males and females. Mean AS was similar within C male groups, but was lower in MOF0ex and MOF1ex males than MO (Figure 4A). Among C female groups, CF1ex AS mean was decreased compared to C and CF0ex. C and CF0ex were similar. In the MO female groups, both F0ex and F1ex intervention reduced mean AS in comparison with the MO group; however, a higher reduction in mean AS was observed in MOF1ex (Figure 4D). AS data failed both normality and homocedasticity tests. Comparison of means alone is therefore an

incorrect approach and is used here only to describe differences in means by one way ANOVA due to the generalized use of this approach in most studies. The scatter plot from C group male and female F1 showed more concentrated data; while male and female F1 from the MO groups show a bigger data spread. In addition, MOF0ex and MOF1ex reduced the data spread in both sexes (Figures 4A,D). A non-parametric comparison of AS showed that median AS was higher in MO when compared to C (*p* < 0.05) for both males and females. Median AS was similar within C male groups while MOF0ex and MOF1ex AS was decreased compared with the MO group (Figures 4B,C). In female C groups, AS was lower in the CF1ex group vs. CF0ex but was similar to C. In MOF0ex and MOF1 females AS was decreased compared with MO females (Figures 4E,F).

**FIGURE 5:**
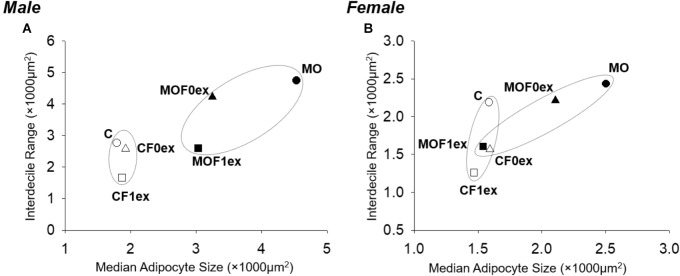
Adipocyte median and interdecile rage in offspring at 110 days. **(A)** Male and **(B)** female adipocyte median and data dispersion (interdecile distance (difference of 90th–10th percentile). C, CF0ex, and CF1ex are represented by open symbols and MO, MOF0ex, and MOF1ex are represented by close symbols.

Among C groups, the plots of AS median vs. Interdecile Rage showed that CF0ex predominantly reduced AS data dispersion in females but not in males (Figure 5). CF1ex mainly reduced data dispersion in males and females; whilst among MO groups, MOF0ex mostly reduced central tendency in both males and females and MOF1ex reduced AS central tendency in males and females; however, data dispersion was only reduced in males.

#### AS Cumulative Distribution Comparisons

**FIGURE 6:**
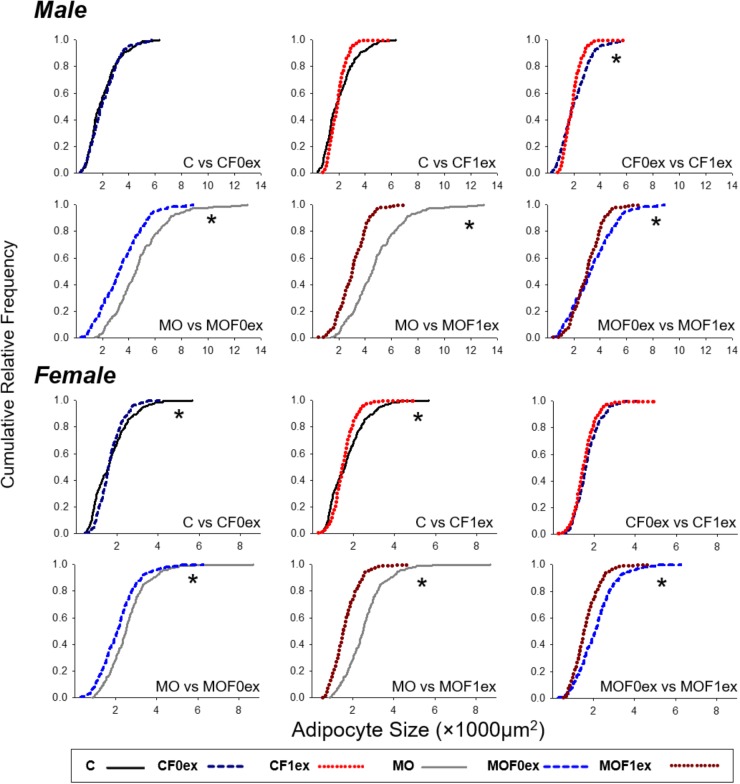
Adipocyte size cumulative distributions in offspring at 110 days. Cumulative distributions were compared by two sample Kolmogorov–Smirnov test and the *p*-Values adjusted by Bonferroni correction for groups with different exercise intervention but the same maternal diet. *n* = 179–250 adipocytes from eight rats per group. ^∗^*p* < 0.05 between the two groups compared.

Male and female C cumulative AS distributions were different (*p* < 0.0001) from MO (Figure 6). CF0ex and CF1ex male AS distributions were different but similar to C. Male MO, MOF0ex and MOF1ex were different (*p* < 0.05). Female CF0ex and CF1ex were similar but different from C. MO, MOF0ex and MOF1ex were different (*p* < 0.05).

#### AS Distribution Modeling

**FIGURE A1:**
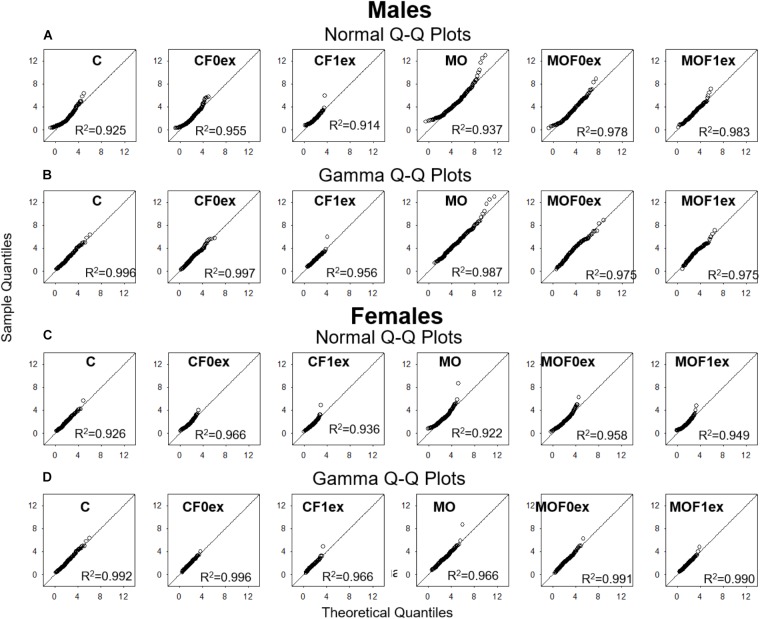
Q-Q plots. Male and Female Normal **(A,C)** and Gamma **(B,D)** theoretical quantiles were plotted against the sample observed quantiles in each group and the coefficient of determination *R*^2^ was calculated in each case to evaluate the normal and gamma distribution as statistical models for adipocyte size.

In all groups, analysis of Q-Q plots showed that AS distribution fits to their theoretical normal and gamma distributions with *R*^2^ values greater than 0.9. However, the linearity in gamma Q-Q plots was greater than normal Q-Q plots, while gamma Q-Q plots showed expected quantile, values were similar to the observed quantile values throughout distribution (Appendix Figure A1).

#### Gamma Distribution and Proportions of Small and Large Adipocytes

**FIGURE 7:**
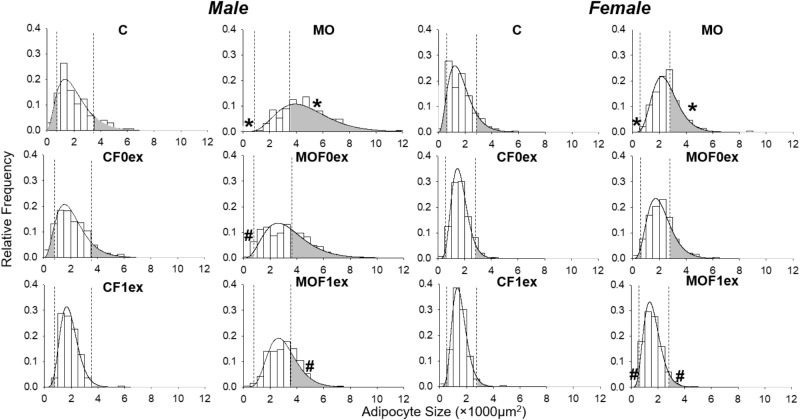
Adipocyte size by gamma distribution. Relative frequency histograms and the gamma distribution function are represented for each maternal group (C, CF0ex, and CF1ex) and (MO, MOF0ex, and MOF1ex). Small and large adipocyte proportions are represented by the shaded region under the curve below and above the respective adipocyte size cut-off points defined by the 10th and 90th percentile in male and female C group; ^∗^*p* < 0.05 vs. C, ^#^*p* < 0.05 vs. MO by non-parametric comparisons of the small and large adipocyte proportions; *n* = 8 per group.

Figure 7 shows the histogram and the gamma distribution function for each group. Small and large adipocyte cut-off points were defined, respectively, by the 10th and 90th percentile of the gamma distribution of the C group, which was 778 μm^2^ for small and 3,587 μm^2^ for large adipocyte in C males and 702 μm^2^ for small and 2,852 μm^2^ for large adipocyte in C females. Decreased small and increased large adipocytes were observed in MO males and females compared to C. Lower proportions of small and large adipocytes were found in male CF1ex compared to CF0ex but similar than C. A higher proportion of small adipocytes was found in male MOF0ex and female MOF1ex compared to MO. A lower proportion of large adipocytes was observed in male and female MOF1ex compared to MO (Figure 7 and Table 1).

**Table 1:** Small and large adipocyte size proportions.

Experimental Group	Male		Female	
	Small adipocytes	Large adipocytes	Small adipocytes	Large adipocytes
	(≤778 μm^2^)	(≥3,587 μm^2^)	(≤702 μm^2^)	(≥2,852 μm^2^)
C	10%	10%	10%	10%


MO	0.1%^∗^	68.6%^∗^	0.4%^∗^	38.9%^∗^


MOF0ex	1.2%^#^	38.5%	2.3%	19.7%


MOF1ex	0.2%	27.1%^#^	4.2%^#^	4.4%^#^


*Small and large adipocyte cut-off points were defined, respectively, by the 10th and 90th percentile of the gamma distribution of the C group and analyses by non-parametric comparisons. ^∗^p < 0.05 vs. C; ^#^p < 0.05 vs. MO; n = 8 per group.*

### Blood Measurements

**FIGURE 8:**
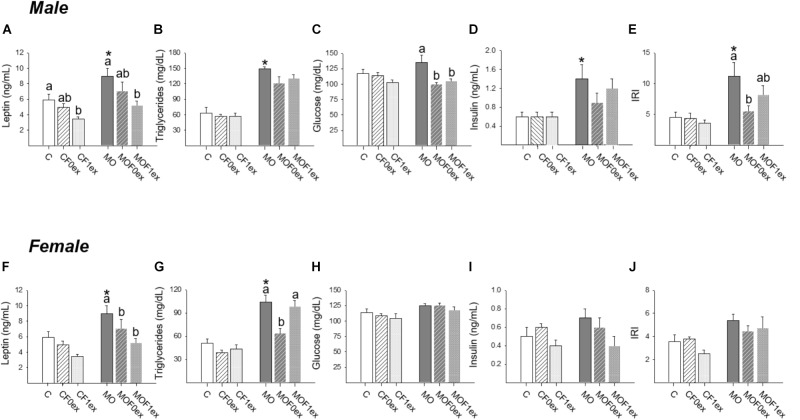
Offspring metabolic parameters. Male and female serum: leptin **(A,F)**, triglycerides **(B,G)**, glucose **(C,H)**, insulin **(D,I)** and insulin resistance index (IRI) **(E,J)**; mean ± SEM, *n* = 8 rats per group; ^∗^*p* < 0.05 vs. MO and for groups not sharing at least one letter (a, b) in the same maternal diet (C, CF0ex, and CF1ex) or (MO, MOF0ex, and MOF1ex).

Increased leptin concentrations were observed in MO males and females compared to C (*p* < 0.05). However, within C and MO males, decreased leptin was observed in CF1ex and MOF1ex (Figure 8A). In C females F0ex and F1ex did not alter leptin concentrations. However, in MO females both MOF0ex and MOF1ex lead to lower leptin concentrations (Figure 8F).

Increased TG concentrations were observed in MO males and females compared to C (*p* < 0.05). Serum TG were similar among male C and MO groups (Figure 8B). In female TG concentrations were similar within C groups. In female MO groups, serum TG concentrations were only reduced in the MOF0ex (Figure 8G).

Male and female serum glucose was similar between C and MO, and within C groups and female MO groups. Male MO glucose was higher than MOF0ex and MOF1ex (*p* < 0.05) (Figures 8C,H). Male but not female MO serum insulin was higher than C (*p* < 0.05); no differences were found among C groups and MO groups in serum insulin (Figures 8D,I). IRI was increased in MO males compared with C (*p* < 0.05). Among C groups F0ex or F1ex did not modify IRI. Among MO groups, IRI was lower in MOF0ex vs. MO, while no difference was observed in the MOF1ex when compared with MO and MOF0ex (Figure 8E). In females, no differences were observed in IRI in any group (Figure 8J).

## Discussion

Metabolic heterogeneity among obese and non-obese individuals, may be related to differences in AS distribution within different adipose tissue compartments (McLaughlin et al., 2014). These differences potentially help explain why individuals with similar total adiposity exhibit dissimilarities in insulin sensitivity (McLaughlin et al., 2004). In the present study, we compared effects on AS of F0 and F1 exercise in young adult F1 of control and obese mothers. The results show that F0 and F1 exercise result in different beneficial metabolic profiles and several dissimilar AS distribution patterns.

It is known that increased AS and number are associated with the manner in which different metabolic states affect adipocyte metabolism (Garaulet et al., 2006; Bjorndal et al., 2011; Fang et al., 2015). Thus, precise methods to identify and quantify adipocyte characteristics are of great scientific and clinical interest. The discrepancy between parametric and non-parametric statistical analyzes of AS and variability indicates a need for development of appropriate methods. For example, methods should include analysis of extreme sizes (very small and large adipocytes). This consideration is often lacking in conventional approaches that consider small and large adipocytes as outliers rather than functionally significant data with important physiological implications. Adipocytes do not divide after preadipocyte differentiation (Scott et al., 1982). Very small AS is associated with preadipocyte differentiation into new adipocytes, whilst large sizes are related to fat accumulation in mature adipocytes (Welte, 2015). The quantification of new adipocytes together with the evaluation of lipid accumulation in mature adipocytes provides a better understanding of metabolic implications of variation in adipose tissue. Fat tissue can be expanded by both hypertrophy and hyperplasia. The balance between these two mechanisms is a major factor determining the final outcome in lipid storage homeostasis. In the present study, we focused on retroperitoneal fat tissue because in rats this fat compartment is expanded by a balance of hyperplasia (58%) and hypertrophy (42%) this is more balanced than other depots. In inguinal fat hyperplasia (65%) predominates while in mesenteric and epididymal depots hypertrophy accounts for 83 and 64%, respectively (DiGirolamo et al., 1998). In addition, increased retroperitoneal fat is associated with glucose and lipid metabolic dysregulation (Matsuzawa et al., 1994; Gabriely et al., 2002).

Adipocyte size shows wide variance and data clustering in the present study since the mean value is larger than the median as a result of an increase in the proportion of large cells. As previously reported, this finding best fits to a gamma distribution (Lenz et al., 2016). Gamma distribution has been used to model waiting times and many other phenomena in social, biological, and physical sciences and is frequently used to describe continuous variables that only take positive values such as, absolute distance, area or volume (Duyckaerts et al., 1994; Reese and Keeley, 2015). The asymmetric distribution of AS is due to the fact that adipocytes that reach a critical size provide signals to neighboring preadipocytes to divide and differentiate (Faust et al., 1978). These paracrine signals can stimulate or inhibit adipogenesis (MacDougald and Mandrup, 2002).

Our results confirm differences between C and MO in leptin, TG, IRI and AS distribution in male and female F1. F0 and F1 exercise conferred different metabolic benefits on F1. The need to undertake more sophisticated analyses of AS is shown by the fact that when using simple median and mean analytic approaches, all groups of male and female controls are similar. However, differences can be shown between C and MO (Samuelsson et al., 2008; Zambrano et al., 2010; Borengasser et al., 2013). The limitation of using this approach is that the two different exercise interventions show similar outcomes compared with their respective controls. However, cumulative distribution analysis demonstrated differences between F0ex and F1ex in C and MO. The novel feature of the gamma distribution analysis we have performed is the evaluation of extreme AS values which can’t be analyzed by median comparisons.

Asymmetry in AS distribution is an important consequence of adipocyte proliferation and differentiation, since small adipocytes are directly linked to an increased rate of adipocyte proliferation and large adipocytes are associated with an increased lipid storage capacity. MO decreased asymmetry compared to C, but MOF0ex increased asymmetry. An asymmetric distribution to greater numbers of small adipocytes as observed in C groups can be interpreted as a better regulation of lipid storage homeostasis than seen in MO. MOF0ex increased the proportion of small cells and increased the proportion of large cells. These changes were accompanied by reduced male IRI and female TG. MO loss of asymmetry was due to both a decrease in small and an increase in large adipocytes which was corrected by both exercise interventions and associated with reduction of F1 serum leptin. In both MO and C, F1ex decreased data dispersion in the presence of lower leptin levels including in the male CF1ex.

Regulation of AS and its distribution in the retroperitoneal adipose tissue as a result of in utero over nutrition likely involves several maternal physiological factors such as preexisting maternal obesity, maternal western diet, and varying degrees of maternal glucose intolerance, which very often are associated to increased offspring birthweight (Desai et al., 2013). As reported previously, voluntary exercise intervention in obese mothers, results in similar F1 birthweight among C and MO of sedentary and exercise mothers, even if F0 MO females at breeding presented higher serum levels of leptin, cholesterol, TG, insulin, glucose, IRI compared to C, and F0 exercise improved them (Vega et al., 2015).

The present study shows that F0 and F1 exercise have positive effects on F1 metabolism that has been programmed by MO. However, the changes that occur in AS are different. F0ex decreases large adipocytes and increases small adipocytes in comparison with MO. F1 exercise in contrast decreases the proportion of large cells more than F0ex but has no effect on the proportion of small cells. One potential explanation is that while exercise occurs during programming in F0ex, in F1 ex the consequences of programming have already been established. However, it is clear that F1 exercise does have beneficial effects. In addition, our data show F1 sexual dimorphism in programming by MO as well as F0 and F1 exercise responses. Men frequently achieve greater body weight and fat mass losses in response to lifestyle interventions than women (Aadland et al., 2014), Thus gender-specific prevention strategies are needed to increase the chance of successful weight loss to prevent or delay the onset of metabolic complication and improve life course health.

## Conclusion

Several factors such as F0 and F1 diet and physical activity and F1 gender modify different aspects of AS distribution. To date outcomes have generally been analyzed by common statistical methods that do not include analyzes of extreme AS which characterize different phenotypes. The differences we have documented in AS distribution likely represent variation in visceral fat adipogenesis regulation, which can be programmed during critical windows of development.

The present study focused on visceral adipose tissue. Similar analyses need to be performed on regulation of AS distribution within other adipose tissue compartments. F0 and F1 exercise were beneficial in F1 metabolism by different mechanism with sex specific effects.

The methods reported here need to be applied in future studies in different experimental species to establish the correlation between AS distribution in visceral adipose tissue and the many specific factors that regulate adipose tissue development and maintenance, e.g., adipokines, glucocorticoids and others steroid hormones (Mohamed-Ali et al., 1998; Sarantopoulos et al., 2017). They will also help determine life course efficacy of interventions designed to prevent and/or recuperate adverse developmental programming outcomes.

## Data Availability Statement

The raw data supporting the conclusions of this manuscript will be made available by the authors, without undue reservation, to any qualified researcher.

## Author Contributions

CI researched the data, designed the study, and wrote the manuscript. MV-M, JL-C, LR-C, GR-G, and CB researched the data. PN prepared the manuscript. EZ designed the study and prepared the manuscript.

## Conflict of Interest Statement

The authors declare that the research was conducted in the absence of any commercial or financial relationships that could be construed as a potential conflict of interest.
